# Hypoxia-induced LINC00674 facilitates hepatocellular carcinoma progression by activating the NOX1/mTOR signaling pathway

**DOI:** 10.7150/jca.76458

**Published:** 2022-08-29

**Authors:** Ning Zhu, Xiaohong Chen, Junjun Zhao, Lijuan Fang, Yingmin Yao, Feifei Zhou, Liang Tao, Qiuran Xu

**Affiliations:** 1 The Key Laboratory of Tumor Molecular Diagnosis and Individualized Medicine of Zhejiang Province, Zhejiang Provincial People's Hospital, Affiliated People's Hospital, Hangzhou Medical College, Hangzhou 310014, China.; 2 Department of Pediatrics, Central Hospital of Haining, Zhejiang Provincial People's Hospital Haining Hospital, Haining 314400, China.; 3 Graduate Department, Bengbu Medical College, Bengbu 233030, China.; 4 Department of Laboratory, Hangzhou Ninth People's Hospital, Hangzhou 310014, China.; 5 Department of Hepatobiliary Surgery, the First Affiliated Hospital of Xi'an Jiaotong University, Xi'an 710061, China.; 6 Department of traditional Chinese Medicine, Zhejiang Provincial People's Hospital, Affiliated People's Hospital, Hangzhou Medical College, Hangzhou 310014, China.; 7 Department of General Surgery, Central Hospital of Haining, Zhejiang Provincial People's Hospital Haining Hospital, Haining 314400, China.

**Keywords:** Hepatocellular carcinoma, Hypoxia, LINC00674, NOX1, mTOR pathway

## Abstract

The hypoxic tumor microenvironment, a fundamental feature of solid tumors, drives hepatocellular carcinoma (HCC) progression through regulating the transcriptional activities of protein-coding and noncoding genes. However, long noncoding RNA (lncRNA)-mediated HCC progression in hypoxic microenvironment remains largely unknown yet. In this study, we found that LINC00674 was upregulated under hypoxic conditions in a HIF-1-dependent manner, and the occupancy of HIF-1 to HRE of LINC00674 gene promoter was essential for its transcription. In addition, LINC00674 level was increased in HCC cell lines and tissues. Clinically, statistical analysis showed that LINC00674 expression was significantly associated with tumor size, venous infiltration, tumor stage and poor prognosis of HCC. Functionally, loss-of-function assays revealed that LINC00674 knockdown inhibited the migration, proliferation and invasion of HCC cells. Furthermore, LINC00674 silencing prominently repressed the mTOR signaling pathway. LINC00674 overexpression-enhanced HCC cell proliferation, migration and invasion were markedly abolished by an mTOR inhibitor rapamycin. NADPH oxidase 1 (NOX1) was positively regulated by LINC00674 in HCC cells. NOX1 knockdown markedly reversed LINC00674-upregulated the p-mTOR level and HCC cells' malignant behaviors. Finally, we found that LINC00674 knockdown attenuated the growth of HCC cells *in vivo*. Our finding demonstrated that LINC00674 was a new HIF-1 target gene, and hypoxia-induced LINC00674 exerted a pro-proliferative and pro-metastatic role in HCC, possibly by activating the NOX1/mTOR signaling pathway. This study suggested LINC00674 as a promising therapeutic target for HCC.

## Introduction

As an increasing global health burden, hepatocellular carcinoma (HCC), with a 5-year survival rate of 18%, accounts for the majority of primary liver cancer 1, 2. Globally, hepatitis B virus (HBV)/HCV infection, alcoholic liver disease and metabolic-dysfunction-associated fatty liver disease (MAFLD) are the leading causes of HCC, and the incidence of HCC continues to increase 3. Though the advances in HCC therapy have been made in the last decades, the general outcome of HCC patients remains unsatisfactory 3. Thus, it is urgent for us to further elucidate the exact molecular mechanisms of HCC.

Hypoxia is a fundamental feature of solid tumors, including HCC 4. Hypoxic tumor microenvironment is caused by increased oxygen consumption due to proliferation and reduced oxygen delivery 5. Hypoxia response is mainly driven by hypoxia inducible factors (HIFs), regulating the transcriptions of hundreds of genes and the activation of signal pathways, such as mitogen-activated protein kinase (MAPK)/ERK pathway, transforming growth factor-β/SMAD family member 3 (TGF-β/SMAD3) pathway and phosphoinositide 3-kinase (PI3K)/AKT/mammalian target of rapamycin (mTOR) pathway and so on 6-8. HIF-1, consisting of HIF-1α and HIF-1β, is the main hypoxia inducible factor 9. HIF-1α, which is stable under hypoxia, forms heterodimer with HIF-1β 9. For HIF-1-mediated gene transcription, the occupancy of HIF-1 at hypoxia response element (HRE) site of the target gene is required 9.

Long noncoding RNAs (lncRNAs) is a kind of non-coding RNAs that have the length of more than 200 nt and have no ability to code proteins 10. Specific patterns of lncRNA expression modulate cancer progression, including HCC 10-12. Based on the findings so far, although most of the hypoxia-related genes are protein-coding genes, more and more lncRNAs have been identified as the HIFs target genes 13. For example, lncHILAR has been identified as a hypoxia-induced gene in renal cancer cells 14. MALAT1 has been identified as a hypoxia-induced lncRNA in breast cancer cells 15. And, RUNX1-IT1, MAPKAPK5-AS1 and KDM4A-AS1 are hypoxia-responsive lncRNAs in HCC 16-18. Thus, lncRNAs involves in hypoxia-enhanced HCC progression. However, the underlying mechanism needs further investigation.

Here, we disclosed the regulatory effect of hypoxia on LINC00674 expression and its underlying mechanism. Next, we revealed the expression, clinical significance and biological function of LINC00674 in HCC. The potential pathways regulated by LINC00674 were investigated by Kyoto Encyclopedia of Genes and Genomes (KEGG) enrichment analysis. Our data showed that LINC00674 was transcriptionally activated by HIF-1 and highly expressed in HCC. LINC00674 contributed to the malignant behaviors of tumor cells, possibly by activating the NADPH oxidase 1 (NOX1)/mTOR signaling pathway in HCC.

## Material and Methods

### Tissue samples

The 75 paired HCC specimens and adjacent non-tumor (NT) tissue samples, were collected from patients in the First Affiliated Hospital of Xi'an Jiaotong University. Inclusion criteria: (1) All patients were pathologically diagnosed with HCC; (2) All of the patients did not receive treatment before surgery; (3) All patients underwent R0 surgical resection; (4) All patients had complete medical records and follow-up data. Exclusion criteria: (1) Combined with other malignant tumors; (2) The patient developed other malignancies during follow-up. All of the samples were stored at -80 °C. The study was conducted according to the guidelines of the Declaration of Helsinki, and approved by the Ethics Committees of the First Affiliated Hospital of Xi'an Jiaotong University (No: XJTU1AF2020LSK-123). Informed consent was obtained from all subjects involved in the study.

### Cell culture

The human embryonic kidney (HEK) 293T cells, human normal liver cell line (L02) and HCC cell lines (Hep3B and MHCC97H) were previously maintained in our lab 19. All of the cells were cultured in DMEM (Gibco, Grand Island, NY, USA) supplemented with 10% FBS (Gibco) and 1% penicillin-streptomycin (Gibco), and maintained in an incubator (37 °C, 5% CO_2_).

### Lentivirus transduction

LKO.1-puro lentiviral vectors encoding small hairpin RNA (shRNA) targeting HIF-1α (sh1α#1 and sh1α#2), NOX1 (shNOX1) or LINC00674 (shLINC#1 and shLINC#2) and non-targeting shRNA (NTC) were obtained from Genechem (Shanghai, China). All lentiviral shuttle vectors were transfected into HEK293T cells for packaging. Lentivirus transduction was performed according to the manufacturers' instructions 20. Puromycin (1 μg/ml) was added to the culture medium of cells transduced with lentivirus for selection of pools of cells expressing the shRNA. LINC00674 expression plasmid was constructed by inserting Cdna into the pcDNA3.1 vector (Invitrogen, Carlsbad, CA, USA). The vectors were transfected into HCC cells using Qiagen Effectene transfection reagent (Valencia, CA, USA).

### RT-qPCR

Isolation of total RNA was conducted by using TRIzol reagent (Invitrogen, Carlsbad, CA, USA) and was reverse-transcribed into Cdna by a Reverse Transcription Kit (Thermo Fisher Scientific, Waltham, MA, USA). Real-time PCR analysis was performed using SYBR Green Premix PCR Master Mix (Roche, Mannheim, Germany) under ABI HT9600 (Applied Biosystems, Foster City, CA, USA). The relative expression level was normalized to 18S Rrna and was calculated by 2^-ΔΔCt^ methods. Primers sequence for LINC00674 and 18S are shown in Supplementary Table 1.

### Chromatin immunoprecipitation (ChIP) assay

Cells were incubated at 20% or 1% O_2_ for 16 hours, cross-linked in 3.7% formaldehyde for 15 minutes, quenched in 0.125 M glycine for 5 minutes, and lysed with SDS lysis buffer. Chromatin was sheared by sonication, and lysates were precleared with salmon sperm DNA/protein A agarose slurry (Millipore, Billerica, MA, USA) for 1 hour and incubated with antibody against HIF-1α (ab228649, Abcam, Cambridge, MA, USA), or IgG (ab171870, Abcam) in the presence of protein A-agarose beads overnight. After serial washing of the agarose beads with low-salt, high-salt, and LiCl buffers, DNA was eluted in 1% SDS with 0.1 M NaHCO_3_, and crosslinks were reversed by addition of 0.2 M NaCl. DNA was purified by phenol-chloroform extraction and ethanol precipitation, and analyzed by Qpcr. Primer sequences for LINC00674-HRE were shown in Supplementary Table 1.

### Transwell assays

For Transwell migration and invasion assays, the cells (2×10^5^) were seed into the 8-μm-pore Transwell inserts (Corning-Costar, Cambridge, MA, USA) containing 200 μl serum-free medium. The lower chambers were added with 800 μl complete culture medium. For detection of invasion ability, Transwell chambers were pre-coated with 15 Μl Matrigel (BD Biosciences, Bedford, MA, USA). After incubation for 24-48 h, cells passed through the membranes were stained with crystal violet (0.1%) and counted.

### Cell proliferation assay

For MTT assay, transfected cells were planted into 96-well plates (2000 cells/ well). Then at 0, 24, 48, and 72h after seeding, MTT (10 Μl/well, Sigma-Aldrich, St. Louis, MO, USA) was added to each well and incubated for 4h at 37 °C. Then, DMSO (100Μl/well) was used to dissolve the crystals. Absorbance was measured at 490 nm by a microplate reader (Thermo Fisher Scientific). The EdU assay was carried out using the Cell-Light™ EdU Apollo®488 *In vitro* Imaging Kit (RIBOBIO, Guangzhou, China) following the manufacturer's protocol, as previously described 19.

### Western blot

Total protein was isolated from cells with RIPA buffer (Beyotime, Shanghai, China). Protein was separated by 10% SDS-PAGE gels, and then transferred to PVDF membranes (Millipore). After being blocked by 5% nonfat milk for 2h, antibodies for HIF-1α (1:1000, ab228649, Abcam), p-Mtor (Ser2448; 1:2000, 67778-1-Ig, Proteintech, Wuhan, China), Mtor (1:5000, 66888-1-Ig, Proteintech), p-p70S6K (Thr389; 1:1000, #9234, Cell Signaling Technology, Beverly, MA, USA), p-4E-BP1 (Thr37/46; 1:1000, #2855, Cell Signaling Technology), NOX1 (1:1000, 17772-1-AP, Proteintech) and β-actin (1:5000, ab8226, Abcam) were used to incubate membranes at room temperature overnight. Then, the membranes were incubated by the HRP-conjugated secondary antibodies (Beyotime). The blots were detected using enhanced chemiluminescence reagent (Millipore).

### *In vivo* experiments

Ten male nude mice (4-5 weeks) were randomly divided into two groups, and 5×10^6^ Hep3B cells stably transfected with NTC or shLINC#1 were injected into the left upper limb of the mice. Tumor volumes were measured once a week. Four-week tumor growth curves were drawn and cervical dislocation killed nude mice to obtain tumors. The xenograft tumors were subjected to RT-Qpcr for LINC00674 expression. Immunohistochemistry (IHC) was carried out using Ki-67 antibody (27309-1-AP, Proteintech) and NOX1 antibody (ab131088, Abcam) in tumor tissues. Animal research was approved by the Institutional Animal Care and Use Committee of Xi'an Jiaotong University.

### Statistical analysis

GraphPad Prism 6.0 (San Diago, CA, USA) was applied to analyze the data. All of the data were presented as mean ± S.D. Statistical methods in this study included Student's *t*-test, one-way ANOVA, Chi-square test, Kaplan-Meier method and log-rank test. Difference with *P<*0.05 was considered to be statistically significant.

## Results

### LINC00674 is upregulated by hypoxia in a HIF-1-dependent manner

**Figure 1:**
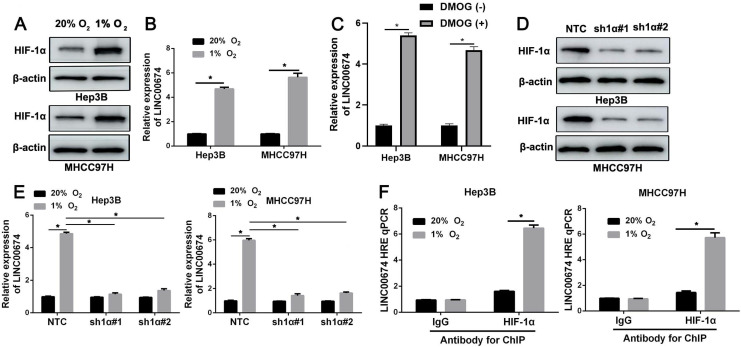
** LINC00674 is induced by hypoxia in a HIF-1-dependent manner. (A)** Hep3B and MHCC97H cells that were exposed to normoxia (20% O_2_) or hypoxia (1% O_2_) for 8 hours were subjected to immunoblotting for HIF-1α. **(B)** Hypoxia upregulated LINC00674 expression in Hep3B and MHCC97H cells. **(C)** DMOG (1mM) treatment increased the expression of LINC00674 in HCC cells. **(D)** The lentivirus mediated shNRAs targeting HIF-1α (sh1α#1 and sh1α#2) and non-targeting shRNA (NTC) were transduced into Hep3B and MHCC97H cells. The transfected cells were exposed to 1% O_2_ for 8 hours, and then western blot was conducted by using antibody against HIF-1α. **(E)** HIF-1α silencing reversed hypoxia-upregulated LINC00674 expression in HCC cells.** (F)** ChIP assays were performed using antibody against HIF-1α or IgG in HCC cells. *P<0.05.

Hep3B cells that were exposed to normoxia (20% O_2_) or hypoxia (1% O_2_) for 24h were analyzed by RNA sequencing. The microarray data (GSE155505) was uploaded into the Gene Expression Omnibus (GEO) 21. LINC00674 was identified as a potential hypoxia-responsive lncRNA. Next, Hep3B and MHCC97H cells were exposed to 20% O_2_ or 1% O_2_ for 24h, and western blot indicated the increased HIF-1α protein (Figure 1A). RT-qPCR analysis revealed that LINC00674 expression was prominently increased in HCC cells under hypoxic conditions (*P*<0.05, Figure 1B and Supplementary Figure 1). Next, HIF prolyl hydroxylase inhibitor (DMOG) induced the upregulation of LINC00674 in HCC cells (*P*<0.05, Figure 1C and Supplementary Figure 2). In order to explore whether LINC00674 was induced by hypoxia in a HIF-1-dependent manner, the HIF-1α was knocked down by two different shRNA constructs in HCC cells (Figure 1D). Our data indicated that the LINC00674 expression was induced by hypoxia, whereas the induction was significantly abrogated by HIF-1α silencing (*P*<0.05, Figure 1E). Furthermore, ChIP assays revealed that hypoxia-induced occupancy of HIF-1α to HRE site of LINC00674 promoter was required for LINC00674 transcription (*P*<0.05, Figure 1F). Moreover, a positive correlation between LINC00674 and HIF-1α mRNA was determined in HCC tissues from the TCGA database using the GEPIA platform (*P*<0.05, Supplementary Figure 3A) 22. Taken together, the above findings demonstrated that LINC00674 was a HIF-1 target gene in HCC cells.

### The expression and clinical significance of LINC00674 in HCC

**Figure 2:**
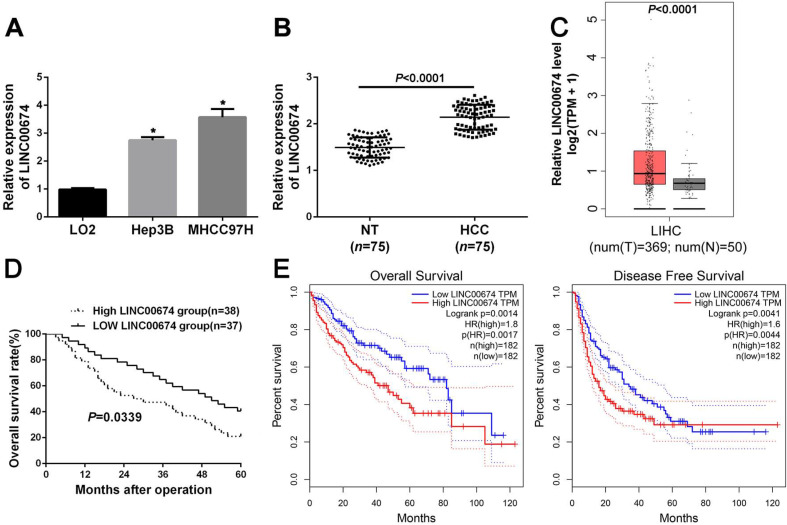
** The expression and clinical significance of LINC00674 in HCC. (A)** The expression of LINC00674 in HCC cell lines (Hep3B and MHCC97H) and normal hepatic cell line L02 were explored by RT-qPCR analysis. **(B)** The levels of LINC00674 in 75 paired HCC and adjacent non-tumor (NT) tissues were explored by RT-qPCR analysis. **(C)** The GEPIA web server was applied to analyze the expression of LINC00674 in HCC (*n*=369) and normal tissues (*n*=50). **(D)** Kaplan-Meier curves were established to explore the predicting value of LINC00674 expression in overall survival rate of HCC patients. **(E)** The GEPIA web server was applied to analyze the prognostic value of LINC00674 expression in HCC. *P<0.05.

Next, the data indicated that LINC00674 expression was significantly upregulated in Hep3B and MHCC97H cells, compared to that in L02 cells (*P*<0.05, Figure 2A). In addition, RT-qPCR analysis revealed that LINC00674 expression in HCC was dramatically higher compared to that in non-tumor tissues (*P*<0.05, Figure 2B), which was consistent with the TCGA data from the GEPIA web server 22 (*P*<0.05, Figure 2C). Based on the median expression of LINC00674 in HCC tissues, the HCC patients were divided into two groups (high/low LINC00674 group). As presented in Table 1, we found that LINC00674 expression was closely correlated to tumor size (*P*=0.004), venous infiltration (*P*=0.017), and TNM stage (*P*=0.014). And higher LINC00674 expression was notably related to worse 5-year overall survival (*P*=0.0339, Figure 2D), which were consistent with the TCGA data from GEPIA web server 22 (*P*<0.05, Figure 2E). Therefore, the above findings demonstrated that overexpressed LINC00674 was a promising marker for prognosis prediction of HCC patients.

### LINC00674 knockdown represses HCC cell proliferation and mobility

**Figure 3:**
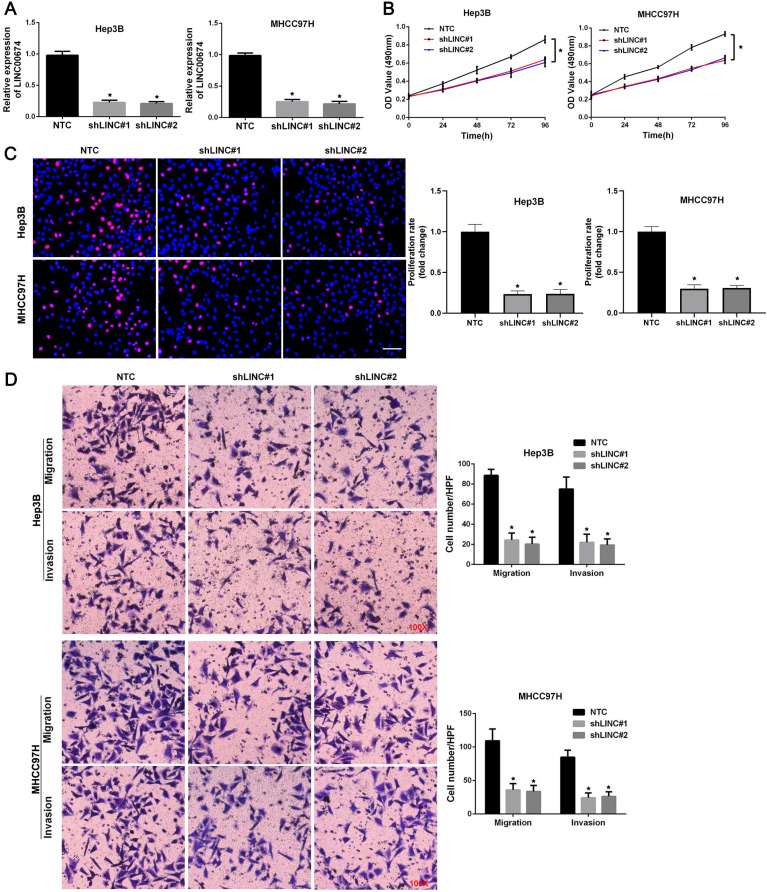
** LINC00674 promotes the malignant behaviors of HCC cells. (A)** The lentivirus mediated shNRAs targeting LINC00674 (shLINC#1 and shLINC#2) and non-targeting shRNA (NTC) were transduced into Hep3B and MHCC97H cells. RT-qPCR analysis was used to confirm the knockdown efficiency. **(B)** MTT assay was used to explore the effect of LINC00674 knockdown on HCC cell viability. **(C)** EdU assay was used to explore the effect of LINC00674 silencing on HCC cell proliferation. Scale bar: 50 µm. **(D)** Transwell assays were used to detect the effect of LINC00674 knockdown on HCC cell migration and invasion. *P<0.05.

To explore the role of LINC00674 in HCC cell migration, proliferation, and invasion, LINC00674 expression was downregulated by two independent shRNA constructs in Hep3B and MHCC97H cells (*P*<0.05, Figure 3A). The results of MTT assay revealed that HCC cell viability was dramatically suppressed by LINC00674 silencing (*P*<0.05, Figure 3B). Consistently, LINC00674 shRNAs significantly inhibited the proliferation of Hep3B and MHCC97H cells, as detected by EdU assay (*P*<0.05, Figure 3C). Then, both transwell migration and invasion assays indicated that the number of Hep3B and MHCC97H cells passing through the membrane was notably decreased by LINC00674 knockdown (*P*<0.05, Figure 3D). Collectively, we demonstrated that LINC00674 exerted a pro-proliferative and pro-metastatic role in HCC cells.

### LINC00674 facilitates the malignant behaviors of HCC cells by activating the mTOR signaling pathway

**Figure 4:**
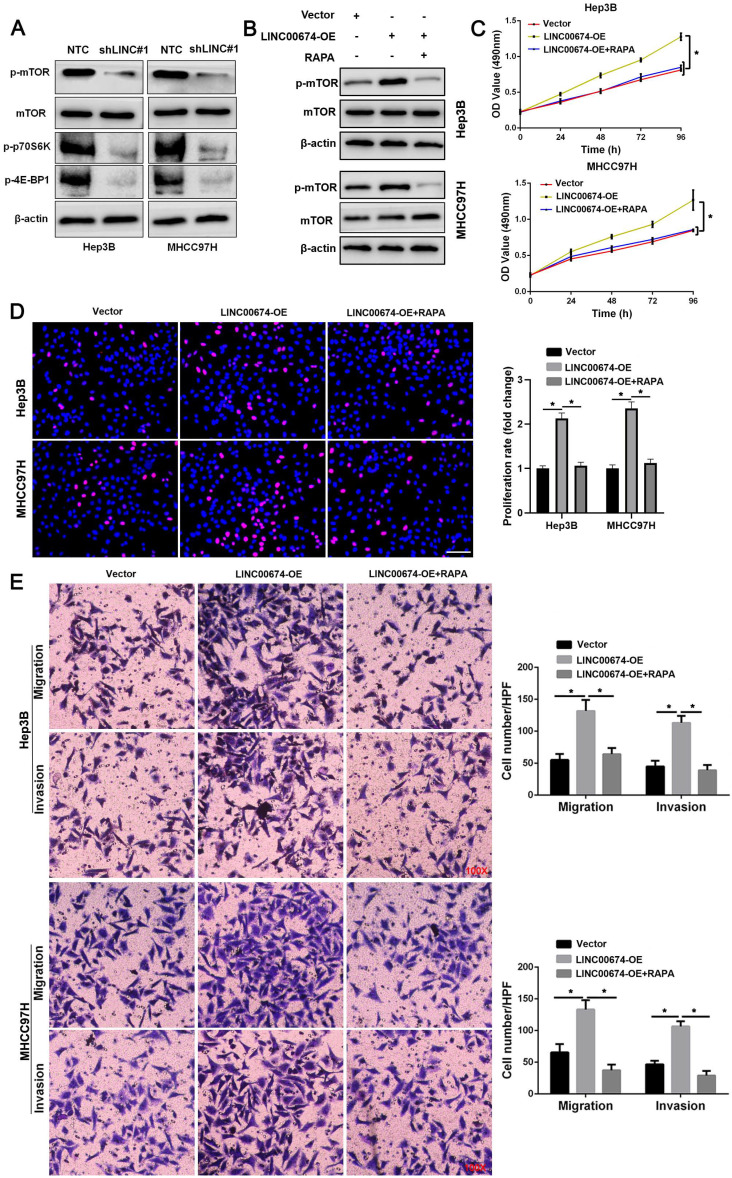
** LINC00674 activates the mTOR signaling pathway in HCC cells. (A)** The lentivirus mediated shNRAs targeting LINC00674 (shLINC#1) and non-targeting shRNA (NTC) were transduced into Hep3B and MHCC97H cells. WB analysis was used to confirm the levels of p-mTOR, p-p70S6K and p-4E-BP1. **(B)** LINC00674 overexpressing HCC cells were treated with RAPA (10 ng/mL) for 48 h, a specific mTOR inhibitor. LINC00674-activated the mTOR signaling pathway was markedly abolished by RAPA treatment. **(C)** MTT, **(D)** EdU, and **(E)** transwell analyses demonstrated that RAPA treatment reversed the tumor-promoting role of LINC00674 in HCC cells. Scale bar: 50 µm for EdU assay. *P<0.05.

KEGG enrichment pathways analysis suggested that LINC00674 might regulate the mTOR signaling pathway in HCC based on TCGA data (Supplementary Figure 4). Western blot data indicated that LINC00674 knockdown reduced the levels of p-mTOR, p-p70S6K and p-4E-BP1 in both Hep3B and MHCC97H cells (Figure 4A). LINC00674 overexpression induced the increased levels of p-mTOR, p-p70S6K and p-4E-BP1 in HCC cells (Figure 4B). Rapamycin (RAPA), an mTOR inhibitor, abolished LINC00674 overexpression-activated the mTOR signaling pathway in HCC cells (Figure 4B). Furthermore, MTT, EdU, and transwell assays showed that LINC00674 overexpression enhanced HCC cell migration, proliferation, and invasion (*P*<0.05, Figure 4C-4E). RAPA treatment remarkably abolished the tumor-promoting role of LINC00674 in HCC cells (*P*<0.05, Figure 4C-4E). Thence, the above findings disclosed that LINC00674 activated the mTOR signaling pathway to promote HCC progression.

### LINC00674 activated the mTOR signaling pathway by enhancing NOX1 expression

**Figure 5:**
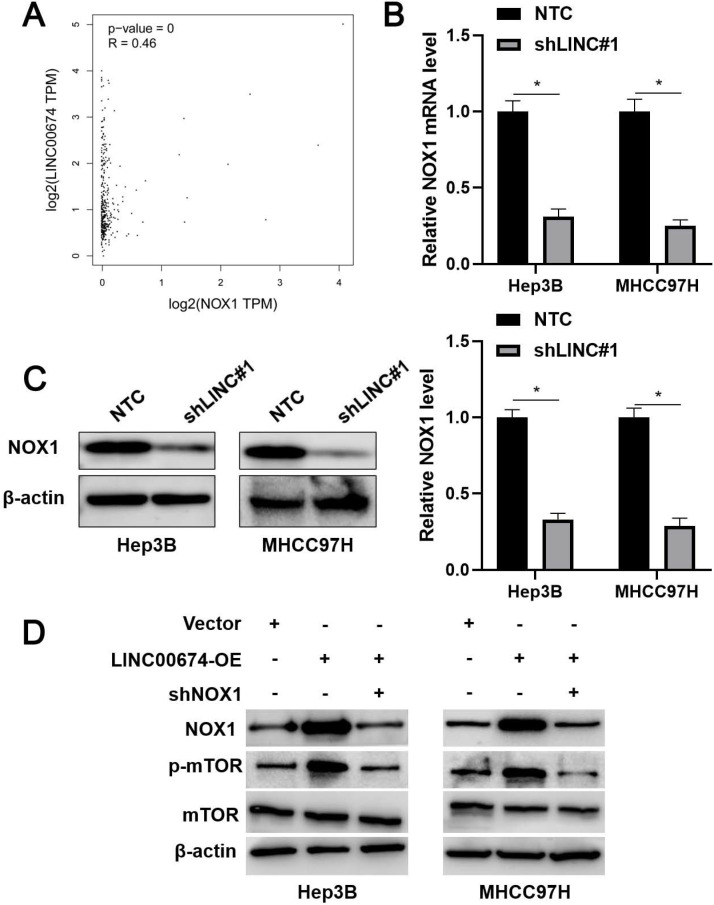
** LINC00674 activates the mTOR signaling pathway by regulating NOX1. (A)** TCGA data analysis by the GEPIA platform indicated a positive correlation between LINC00674 and NOX1 mRNA levels in HCC tissues. **(B and C)** The lentivirus mediated shNRA targeting NOX1 and non-targeting shRNA (NTC) were transduced into Hep3B and MHCC97H cells. WB and RT-qPCR analysis was used to confirm NOX1 mRNA and protein levels. **(D)** LINC00674-activated the mTOR signaling pathway was markedly attenuated by NOX1 knockdown in HCC cells. *P<0.05.

**Figure 6:**
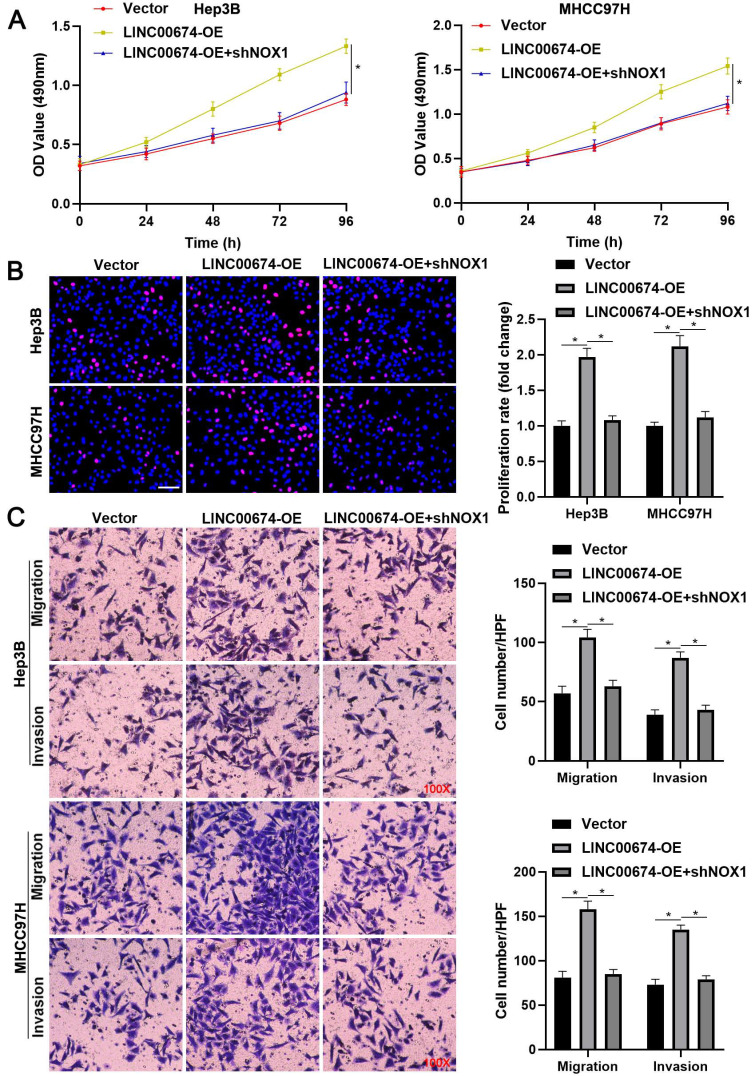
** NOX1 mediates the tumor-promoting role of LINC00674 in HCC cells. (A)** MTT, **(B)** EdU, and **(C)** transwell analyses demonstrated that NOX1 knockdown remarkably abolished the tumor-promoting role of LINC00674 in HCC cells. Scale bar: 50 µm for EdU assay. *P<0.05.

TCGA data analysis using the GEPIA platform 22 indicated that LINC00674 was positively correlated with NOX1 mRNA expression in HCC tissues (*P*<0.05, Figure 5A). Next, we found that LINC00674 knockdown significantly reduced NOX1 mRNA and protein levels in Hep3B and MHCC97H cells (*P*<0.05, Figure 5B and 5C). Notably, NOX1 silencing prominently abolished LINC00674 overexpression-upregulated p-mTOR level in HCC cells (Figure 5D). Notably, NOX1 knockdown remarkably abolished the tumor-promoting role of LINC00674 in HCC cells (*P*<0.05, Figure 6A-6C). Interestingly, NOX1 mRNA was positively correlated with HIF-1α mRNA in HCC tissues from the TCGA database using the GEPIA platform (*P*<0.05, Supplementary Figure 3B) 22. Thus, we suggested that LINC00674 activated the mTOR signaling pathway and facilitated tumor progression by enhancing NOX1 expression in HCC cells.

### LINC00674 silencing attenuates HCC growth *in vivo*

**Figure 7:**
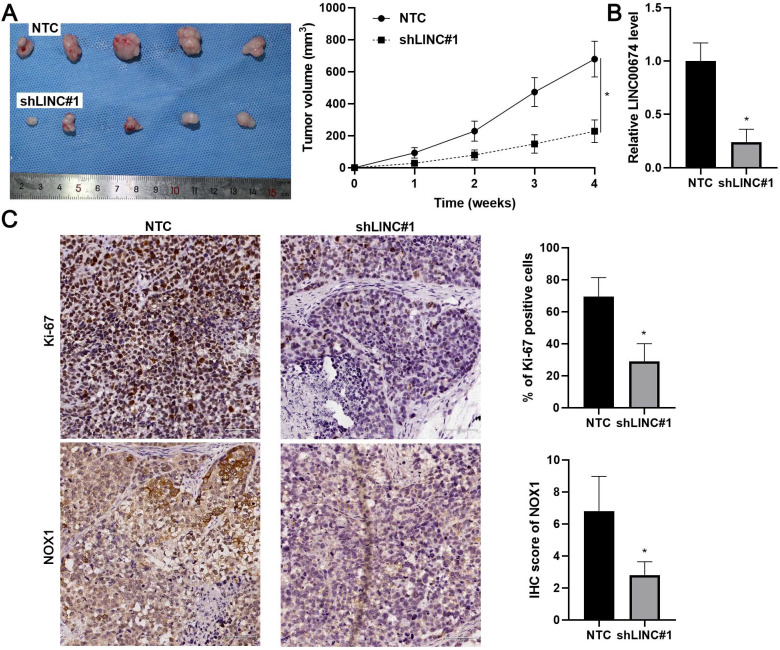
** LINC00674 knockdown attenuates HCC growth in mice. (A)** Hep3B cells without (NTC) or with LINC00674 knockdown (shLINC#1) were subcutaneously injected into nude mice. Four-week tumor growth curves were drawn. **(B)** RT-qPCR assay was performed to detect the LINC00674 level in the xenograft tumor tissues. **(C)** IHC was carried out to determine the levels of Ki-67 and NOX1 in the xenograft tumor tissues. scale bar: 60 µm.

Next, we intended to confirm our findings using the nude mouse subcutaneous tumor implantation experiment. As expected, LINC00674 knockdown prominently attenuated the growth of Hep3B cells *in vivo* (*P*<0.05, Figure 7A). RT-qPCR assay confirmed the lower levels of LINC00674 in tumor tissues collected from the LINC00674 knockdown group (*P*<0.05, Figure 7B). Moreover, the staining of Ki-67 and NOX1 in tumor tissues obtained from the LINC00674 knockdown group was significantly weaker than the control group (*P*<0.05, Figure 7C). Altogether, LINC00674 promoted HCC progression.

## Discussion

As a common feature for solid tumor, hypoxia is closely related to metastasis, growth, angiogenesis, metabolism, therapeutic resistance and immune escape 23. HIFs, which is represented by HIF-1, take charge of the transcriptional activities of hundreds of genes 24. Although among of these HIFs target genes, most are protein-coding genes, growing number of non-coding RNAs have been identified 25. In this study, we identified a novel HIF-1-targeting lncRNA LINC00674. LINC00674 showed increased expression level in HCC cells induced by hypoxia. Most hypoxia-related genes were directly regulated by HIF-1 under hypoxia. We attempted to investigate whether LINC00674 was induced by hypoxia in a HIF-1-dependent manner. As expected, LINC00674 was induced by hypoxia, but the induction was abrogated by HIF-1α knockdown. These results suggest that HIF-1 is essential for the LINC00674 upregulation under hypoxia. Additionally, the data from ChIP assay further confirmed our hypothesis. Although HIF-1 plays the main role in regulating gene transcription, some previous studies also show that some genes can be regulated by HIF-2 26, 27. Thus, further study is required to explore whether LINC00674 is also regulated by HIF-2 in HCC cells under hypoxia.

Next, data from our tissue samples and public datasets showed that LINC00674 expression was increased in HCC. The clinical significance of LINC00674 in HCC was explored. LINC00674 overexpression was closely related to large tumor, venous infiltration, advanced tumor stage and unfavorable prognosis of HCC patients. These findings suggest LINC00674 as a valuable biomarker for HCC. LncRNAs that play important roles in HCC tumorigenesis and progression, provide new insights into the tumor target therapy. Recently, numerous lncRNAs have been reported to be involved in HCC progression, such as CASC2, MCM3AP-AS1, MAPKAPK5-AS1 and RUNX1-IT1 16, 17, 19, 28. In these studies, lncRNAs are involve in the migration, invasion, proliferation, epithelial-mesenchymal transition (EMT), and stemness of HCC cells 16, 17, 19, 28. Here, we found that LINC00674 facilitated the migration, proliferation, and invasion of HCC cells and LINC00674 knockdown attenuated HCC growth in mice. These results suggest that LINC00674 acts as an oncogene in HCC progression.

The KEGG pathway enrichment analysis suggested that LINC00674 expression was correlated with the mTOR signaling pathway in HCC based on TCGA data. Studies have shown that the activation of mTOR signaling pathway is frequently observed in HCC progression 29, 30. mTOR forms a catalytic subunit of two distinct protein complexes, mTOR complex 1 (mTORC1) and mTORC2 31. Activation of mTORC1 most prominently results in phosphorylation of two downstream targets, p70S6K and 4EBP1, promoting protein synthesis 32. 4EBP1 and p70S6K axes play critical and distinct roles in hepatocarcinogenesis 33. Here, we found that LINC00674 knockdown reduced the levels of p-mTOR, p-p70S6K and p-4E-BP1 in HCC cells. The ectopic expression of LINC00674 activated the mTOR signaling pathway. And the rescue experiments revealed that RAPA, an mTOR inhibitor, prominently abrogated LINC00674-induced the mTOR signaling pathway activation and HCC cells' malignant behaviors. Our data indicated that LINC00674 activated the mTOR signaling pathway to promote HCC progression. To explore the mechanism by which LINC00674 increased p-mTOR level in HCC cells, we screened similar genes with LINC00674 in TCGA data based on the GEPIA platform 22. We found a positive correlation of LINC00674 and NOX1 mRNA levels in HCC tissues. LINC00674 silencing prominently reduced NOX1 expression in HCC cells. NOX1 is highly expressed and has been recognized as a tumor-promoting gene in HCC 34. NOX1-mediated ROS production activates the mTOR signaling pathway and enhances tumor growth and metastasis 34, 35. Notably, NOX1 knockdown attenuated LINC00674 overexpression-increased p-mTOR level in HCC cells. Notably, NOX1 knockdown remarkably abolished the tumor-promoting role of LINC00674 in HCC cells. Thus, we suggested that LINC00674 activated the mTOR signaling pathway by enhancing NOX1 expression in HCC cells.

Taken together, our findings identified LINC00674 as a novel HIF-1 target gene in HCC. Hypoxia-induced LINC00674 promoted the growth and metastasis of HCC, possibly by activating the NOX1/mTOR signaling pathway. This study provided LINC00674 as a promising therapeutic target for HCC.

## Supplementary Material

Supplementary figures and table.

## Figures and Tables

**Table 1:** Correlations between LINC00674 expression and the clinicopathologic characteristics of patients with HCC

Characteristics	*n* = 75	LINC00674 expression		*P*
		High	Low	
**Age (year)**				0.276
<50	22	9	13	
≥50	53	29	24	
**Gender**				0.141
Male	62	29	33	
Female	13	9	4	
**HBV**				0.268
Absent	9	3	6	
Present	66	35	31	
**Serum AFP level (ng/mL)**				0.286
<400	16	10	6	
≥400	59	28	31	
**Tumor size (cm)**				0.004
<5	32	10	22	
≥5	43	28	15	
**Number of tumor nodules**				0.166
1	60	28	32	
≥2	15	10	5	
**Cirrhosis**				0.054
Absent	19	6	13	
Present	56	32	24	
**Venous infiltration**				0.017
Absent	51	21	30	
Present	24	17	7	
**Edmondson-Steiner grading**				0.110
I + II	48	21	27	
III + IV	27	17	10	
**TNM stage**				0.014
I + II	53	22	31	
III + IV	22	16	6	

HCC, hepatocellular carcinoma; HBV, hepatitis B virus; AFP, alpha-fetoprotein; TNM, tumor-node-metastasis.

## Abbreviations

HCC: hepatocellular carcinoma
HBV: hepatitis B virus
MAFLD: metabolic-dysfunction-associated fatty liver disease
HIFs: hypoxia inducible factors
HRE: hypoxia response element
lncRNAs: long noncoding RNAs
KEGG: Kyoto Encyclopedia of Genes and Genomes
EMT: epithelial-mesenchymal transition
mTORC1: mTOR complex 1
