# Supplementary material for: Hypoxia-induced LINC00674 facilitates hepatocellular carcinoma progression by activating the NOX1/mTOR signaling pathway
Source: J Cancer. 2022 Aug 29;13(11):3177–88. doi: 10.7150/jca.76458 (PMC9475361; doi:10.7150/jca.76458)
Supplement: Supplementary file 1 — Supplementary figures and table. [file jcav13p3177s1.pdf]

Supplementary Table 1 The primer sequences used in this study

Gene	Primer	Sequence (5′-3′)
LINC00674	forward	GAGCACGTCCACATAGCTGA
	reverse	TGGACTCCTAGCAGGAGCAT
NOX1	forward	GTTTTACCGCTCCCAGCAGAA
	reverse	GGATGCCATTCCAGGAGAGAG
18S	forward	CGGCGACGACCCATTCGAAC
	reverse	GAATCGAACCCTGATTCCCCGTC
HRE-1	forward	TGTTTTCTGGCTGGACACAG
	reverse	CCACCACACCTGGCTAACTT
HRE-2	forward	CTTAAATTGTGCCACCACCA
	reverse	GGCTGAGGAAAGTGAGCCTA

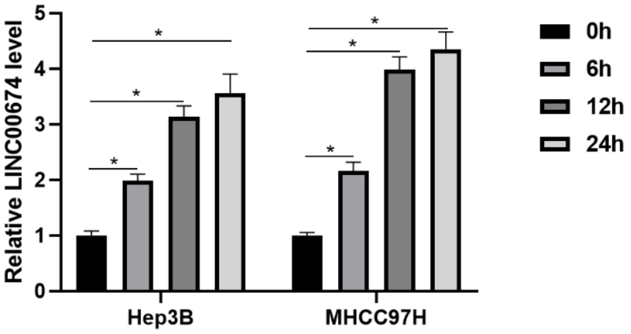

Supplementary Figure 1 The level of LINC00674 in Hep3B and MHCC97H cells under hypoxic conditions at different time points.

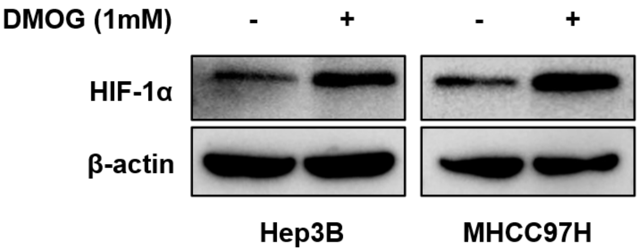

Supplementary Figure 2 The level of HIF-1α in Hep3B and MHCC97H cells with or without DMOG (1mM) treatment.

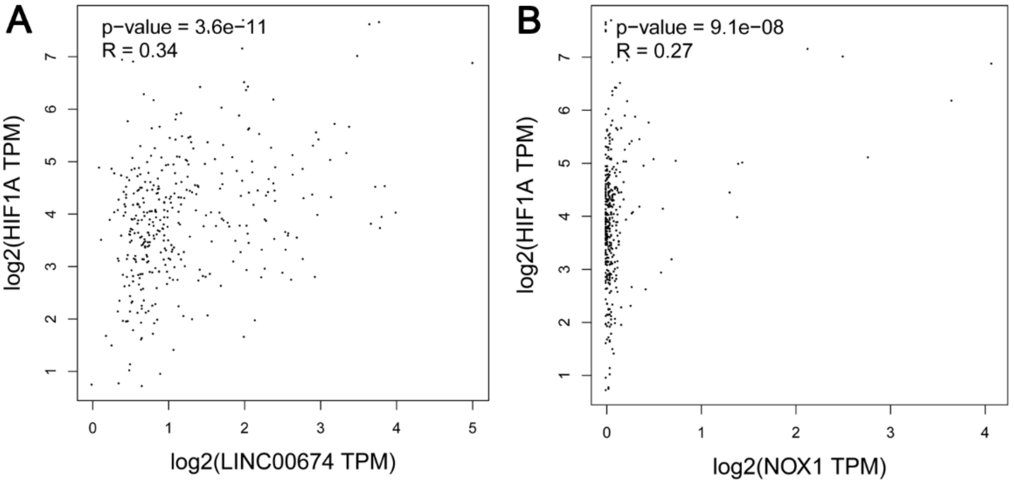

Supplementary Figure 3 The correlations between HIF-1α mRNA and LINC00674/NOX1 mRNA in HCC tissues from TCGA database. (A) HIF-1α mRNA was positively correlated with LINC00674 expression in HCC tissues. (B) HIF-1α mRNA was positively correlated NOX1 mRNA level in HCC tissues.

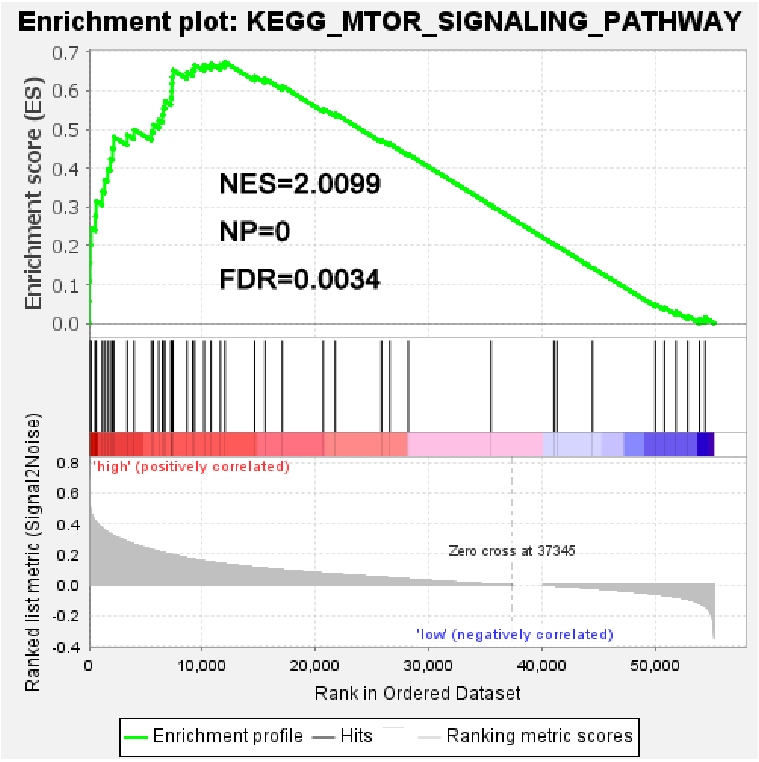

Supplementary Figure 4 KEGG pathway enrichment analysis indicates a close correlation between LINC00674 and the mTOR signaling pathway in HCC.
